# How do environment-dependent switching rates between susceptible and persister cells affect the dynamics of biofilms faced with antibiotics?

**DOI:** 10.1038/s41522-018-0049-2

**Published:** 2018-03-15

**Authors:** Gabriel Carvalho, Damien Balestrino, Christiane Forestier, Jean-Denis Mathias

**Affiliations:** 1 UR LISC Laboratoire d’Ingénierie pour les Systèmes Complexes, Irstea, Aubière, France; 2 LMGE, UMR6023 CNRS, Université Clermont Auvergne, Clermont-Ferrand, France

## Abstract

Persisters form sub-populations of stress-tolerant cells that play a major role in the capacity of biofilms to survive and recover from disturbances such as antibiotic treatments. The mechanisms of persistence are diverse and influenced by environmental conditions, and persister populations are more heterogeneous than formerly suspected. We used computational modeling to assess the impact of three switching strategies between susceptible and persister cells on the capacity of bacterial biofilms to grow, survive and recover from antibiotic treatments. The strategies tested were: (1) constant switches, (2) substrate-dependent switches and (3) antibiotic-dependent switches. We implemented these strategies in an individual-based biofilm model and simulated antibiotic shocks on virtual biofilms. Because of limited available data on switching rates in the literature, nine parameter sets were assessed for each strategy. Substrate and antibiotic-dependent switches allowed high switching rates without affecting the growth of the biofilms. Compared to substrate-dependent switches, constant and antibiotic-dependent switches were associated with higher proportions of persisters in the top of the biofilms, close to the substrate source, which probably confers a competitive advantage within multi-species biofilms. The constant and substrate-dependent strategies need a compromise between limiting the wake-up and death of persisters during treatments and leaving the persister state fast enough to recover quickly after antibiotic-removal. Overall, the simulations gave new insights into the relationships between the dynamics of persister populations in biofilms and their dynamics of growth, survival and recovery when faced with disturbances.

## Biofilm persistence: Strategies for switching

Computer simulations yield useful insights into strategies bacteria in biofilms use to switch into antibiotic-tolerant “persister” states. A diverse range of mechanisms are available to promote the transition into persister forms. The populations of cells in persister states are proving to be more varied than previously thought. Researchers in France, led by Gabriel Carvalho at the Complex Systems Engineering Laboratory in Aubière, modelled different switching strategies that allow biofilms to survive, recover and grow despite antibiotic treatments. Their procedure examines the effect on switching rates of a greater range of variable factors than previous simulation methods. These variables include the concentration and flow patterns of relevant chemical compounds, changing environmental conditions, and the challenge presented by different antibiotics. The results will help research toward understanding and treating a variety of medically significant biofilm infections.

## Introduction

Living as biofilms enables bacterial populations to withstand harsh disturbances such as antibiotic treatments and this lifestyle is the root of many chronic infections and bacterial survival in natural environments.^1–3^ Biofilm survival capacity is often conditioned by the formation of internal sub-populations of persisters, i.e., phenotypic variants tolerant to various stresses such as antibiotics.^4–6^ Unlike resistance, tolerance is temporary and reversible.^7^ A commonly accepted model of bacterial persistence is that a few cells can switch to the persister state in an isogenic bacterial population and, inversely, persisters can switch back to the susceptible state.^8^ Although persisters tolerate stresses, their growth rate is impaired in comparison to that of actively growing susceptible cells. Several mechanisms are involved in the induction of the persister state including toxin/antitoxin modules and stress-responses such as the stringent and the SOS responses.^9–12^ Persisters can be produced randomly or in response to environmental conditions such as nutrient deprivation, heat, extreme pH, quorum-sensing molecules and the presence of antibiotics.^5,9,13^ Persister populations are broadly heterogeneous and bacterial persistence relies on several genes.^14–17^ In addition, persisters are stress-specific: persisters surviving one kind of stress may not survive another kind.^18,19^ As a result, experimental studies on persisters are largely affected by the environmental conditions, strains and stresses used. The intrinsic heterogeneity of biofilms makes these environmental conditions difficult to predict and control at a cellular level within the community. Computational models are useful to simulate this heterogeneity and predict the population dynamics of susceptible and persister cells in biofilms.

A few mathematical biofilm models have been developed with various switching rates between persisters and susceptible cells.^20–24^ Most models assess one switching strategy with one set of parameters, and simulations are seldom correlated with experimental data. Switching rates are often assumed to be low but experimental evidence is scarce.^8,18,25^ Given the heterogeneity of persister populations and persister formation mechanisms it is therefore difficult to compare simulated and experimental biofilms. Instead of differential equations, Chihara et al.^26^ implemented different persister formation strategies in an individual-based model (IBM) of biofilm. This kind of model has been used for more than a decade to simulate biofilm formation, structure and heterogeneity.^27–30^ In IBMs of biofilms, each cell is an independent virtual entity that evolves in a continuous space and interacts with other cells and its microenvironment, such as the local substrate and antibiotic molecules. Using an IBM, Chihara et al.^26^ succeeded in identifying spatial patterns of persister formation in simulated biofilms. However, they did not implement switches from the persister state to the susceptible state nor considered antibiotic treatments. Their model therefore needs to be modified to achieve simulation of biofilm recovery after antibiotic shocks.

**Fig. 1:**
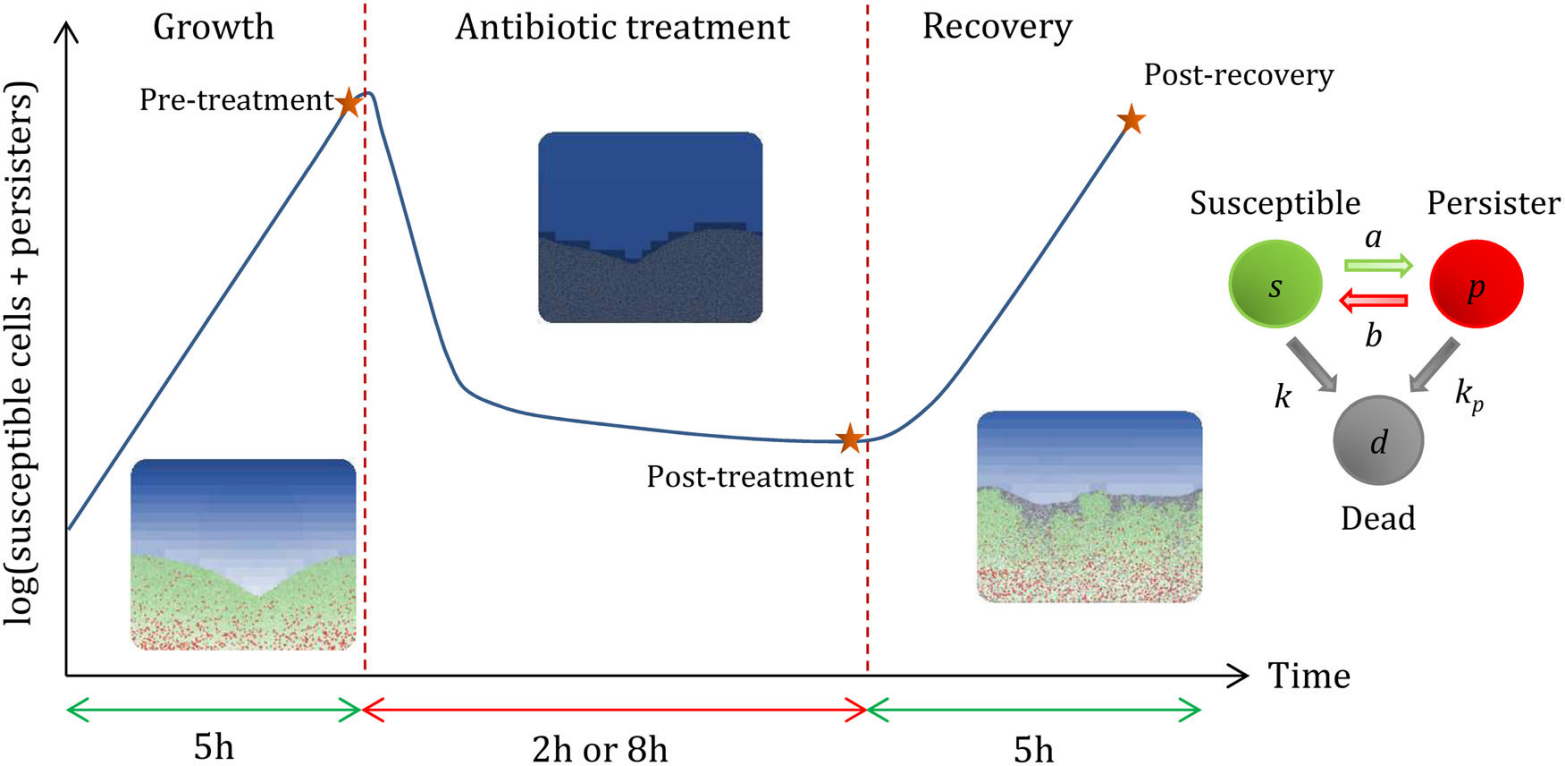
Simulation process. Biofilms grown for 5 h were then treated for 2 or 8 h and allowed to recover during another 5 h after antibiotic removal. Cells can switch between susceptible and persister phenotypes or be killed by the antibiotic. During treatments, susceptible cells die quickly and only persisters survive (*k* » *k*_p_). After antibiotic treatments, persisters that switch back to actively growing susceptible cells allow the biofilm to recover. To assess the effect of the switching strategies on the growth, survival and recovery of the biofilm, we focused on three time points: pre-treatment, post-treatment and post-recovery

The objective of this work was to study the effect of the switching dynamics between susceptible and persister cells on the capacity of biofilms to grow, survive and recover from antibiotic shocks. Because of the diversity of persister producing mechanisms, we tested three switching strategies: (1) constant switches, (2) substrate-dependent switches and (3) antibiotic-dependent switches. There is a paucity of evidence about the maximum rates of these switches and so we postulated that they could be low or high and tested nine parameter sets for each strategy. In the first strategy tested, each cell had a constant probability to become persister or susceptible, regardless of its microenvironment. In the second strategy, persister production was triggered by the lack of substrate and reversion to the susceptible state was triggered by its presence. In the third strategy, persister production was triggered by the presence of antibiotic and the reversion to the susceptible state was triggered by its absence. These strategies were implemented in a two-dimensional (2D) individual-based biofilm model. Persisters were non-growing dormant cells much less affected by antibiotics than actively growing susceptible cells. Population dynamics and structures of treated biofilms were obtained from simulations. Strategies were compared at three time points, pre-treatment, post-treatment and post-recovery, to assess the capacity of the biofilms to grow, survive and recover (Fig. 1). A local sensitivity analysis was also carried out to assess the influence of the model parameters on the different switching strategies.

## Results

### Unlike constant switches, substrate and antibiotic-dependent switches do not impair the fitness of bacterial populations

**Fig. 2:**
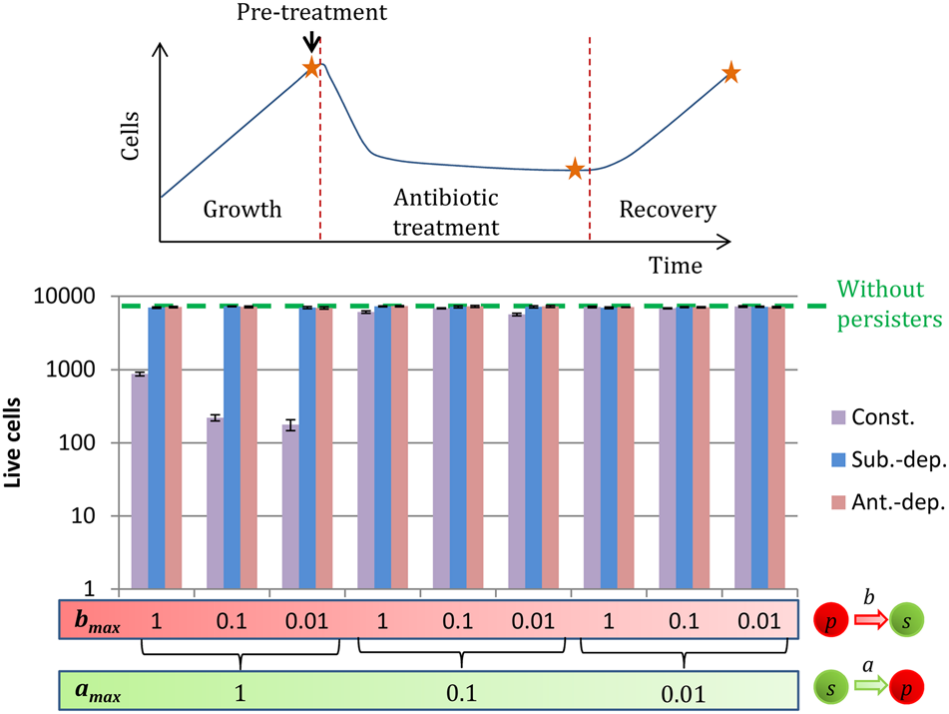
Live cells (susceptible cells *s* + persisters *p*) after 5 h of biofilm formation for the three switching strategies: constant (Const.), substrate-dependent (Sub.-dep.) and antibiotic-dependent (Ant.-dep.). *a*_max_ is the maximum switching rate from the susceptible state to the non-growing persister state. *b*_max_ is the maximum switching rate from the persister state to the susceptible state

For each strategy, nine combinations of the maximum switching rates were tested, from susceptible to persister, *a*_max_, and from persister to susceptible, *b*_max_. With constant switches, high *a*_max_ values significantly impaired the fitness of the bacterial populations. The biofilms obtained after 5 h of formation were much smaller than without switches (Fig. 2). With this strategy, the switching rates were always maximum regardless of environmental conditions, and susceptible cells became non-growing persisters instead of dividing. As a result, the maximum switching rate *a*_max_ had to be low to not impair the fitness of the bacterial population. With substrate-dependent switches, persister formation was maximum in substrate-deprived zones. Susceptible bacteria intended to become persisters had slow growth rates and did not participate in the overall growth of the biofilms. Consequently, high *a*_max_ values led to high quantities of persisters within the biofilms without affecting overall growth (Fig. 2). In contrast, substrate deprivation was needed to facilitate the formation of persisters. Hence, there were almost no persisters within young biofilms, when there is still plenty of substrate and no substrate concentration gradients (<3 h, see Supplementary Information). With antibiotic-dependent switches, persister formation was induced by the antibiotic itself. There was no effect therefore on the fitness of the bacterial population since there was no phenotypic switch in the absence of antibiotic. The capacity to sense environmental conditions, i.e. to sense substrate or antibiotic concentrations, allowed bacterial biofilms to have high maximum rates of persister formation (*a*_max_) without affecting their overall growth rate.

### Only antibiotic-dependent switches prevent the wake-up and death of persisters during treatments

We simulated biofilm formation for 5 h, and then applied antibiotic treatment for 2 and 8 h. Without persisters, the biofilms were eradicated within 2 h after the addition of antibiotic. The constant and substrate-dependent strategies were both greatly affected by the treatment duration, particularly when *b*_max_ was high. With constant switches, persisters switched their phenotype regardless of their environment and woke up despite the presence of antibiotics. With substrate-dependent switches, susceptible cells died quickly during treatment and substrate consumption fell. As a result, the substrate concentration in the biofilms increased and triggered the wake-up of persisters. With these two strategies, when *b*_max_ = 1, the biofilms recovered from the 2 h treatments but all persisters woke up and died during the 8 h treatments. *b*_max_ had to be small for these two strategies to limit the wake-up and death of persisters during treatments. With antibiotic-dependent switches, the antibiotic inhibited the wake-up of persisters. High *b*_max_ values could be used without affecting the survivability of the biofilm cells undergoing antibiotic treatment. Thus, the biofilms were not much affected by the duration of the treatments. The death of persisters was mainly caused by direct killing by the antibiotic (parameter *k*_p_).

**Fig. 3:**
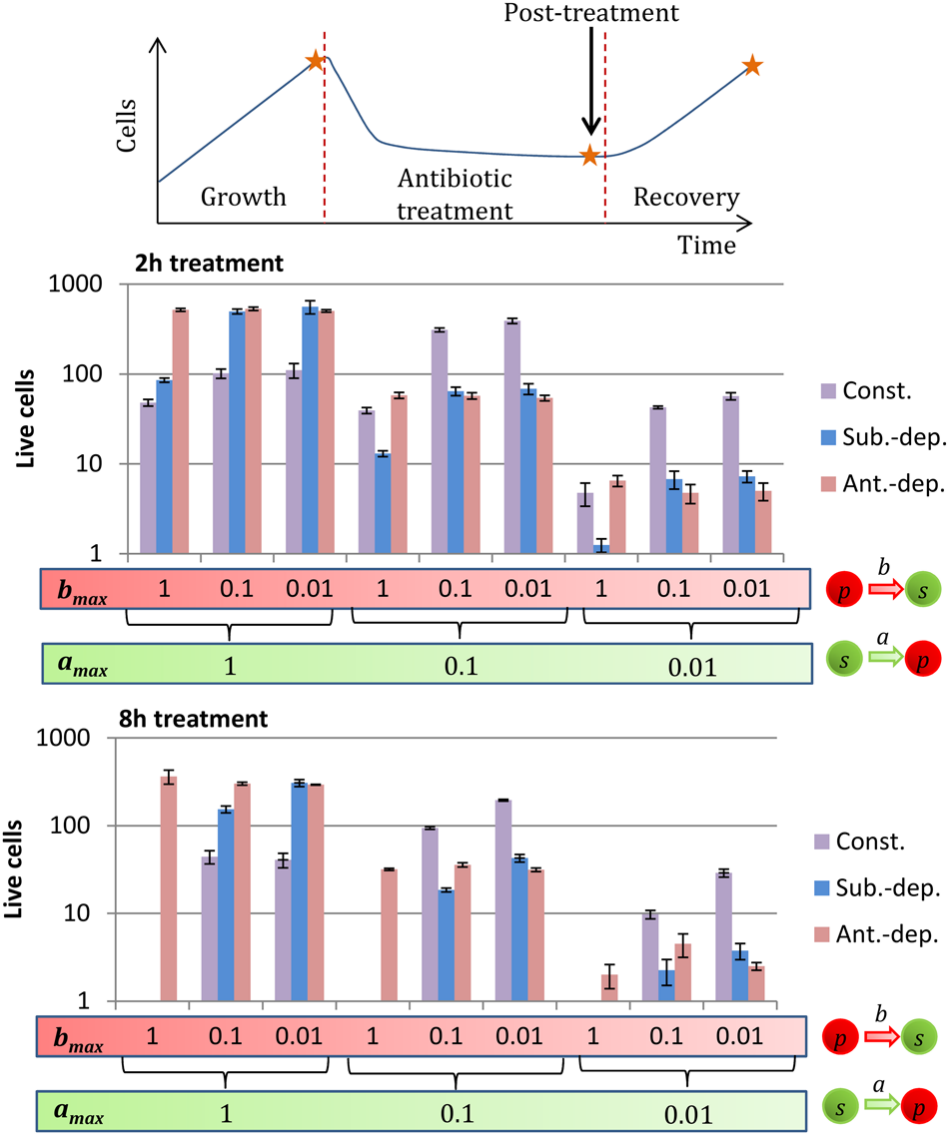
Number of cells surviving after 2 h and 8 h-long antibiotic treatments for the three switching strategies: constant (Const.), substrate-dependent (Sub.-dep.) and antibiotic-dependent (Ant.-dep.). Cells reverting to the susceptible state during treatments died quickly. The populations post-treatment were composed solely of persisters: (*s* + *p*)_post-treatment_ ≈ *p*_post-treatment_. *a*_max_ is the maximum switching rate from the susceptible state to the non-growing persister state. *b*_max_ is the maximum switching rate from the persister state to the susceptible state

We considered the number of cells able to survive an antibiotic treatment (Fig. 3). For the substrate-dependent strategy, the highest quantities of survivors were obtained with high *a*_max_ and small *b*_max_ values, which maximized the production of persisters and limited their wake-up during treatment. With the constant strategy, the intermediate *a*_max_ = 0.1 led to the highest quantities of survivors since this strategy need a compromise between not impairing growth and producing persisters. Constant switches with high *a*_max_ values led to small biofilms with a high proportion of persisters. With antibiotic-dependent switches, susceptible cells must become persisters otherwise they die during treatment. Thus, the quantity of survivors depended on how fast susceptible cells became persisters. The highest quantity of survivors was obtained with the highest *a*_max_ values and, as stated previously, were not much affected by *b*_max_.

### Recovery efficiency depends on the number of persister switches after antibiotic removal

**Fig. 4:**
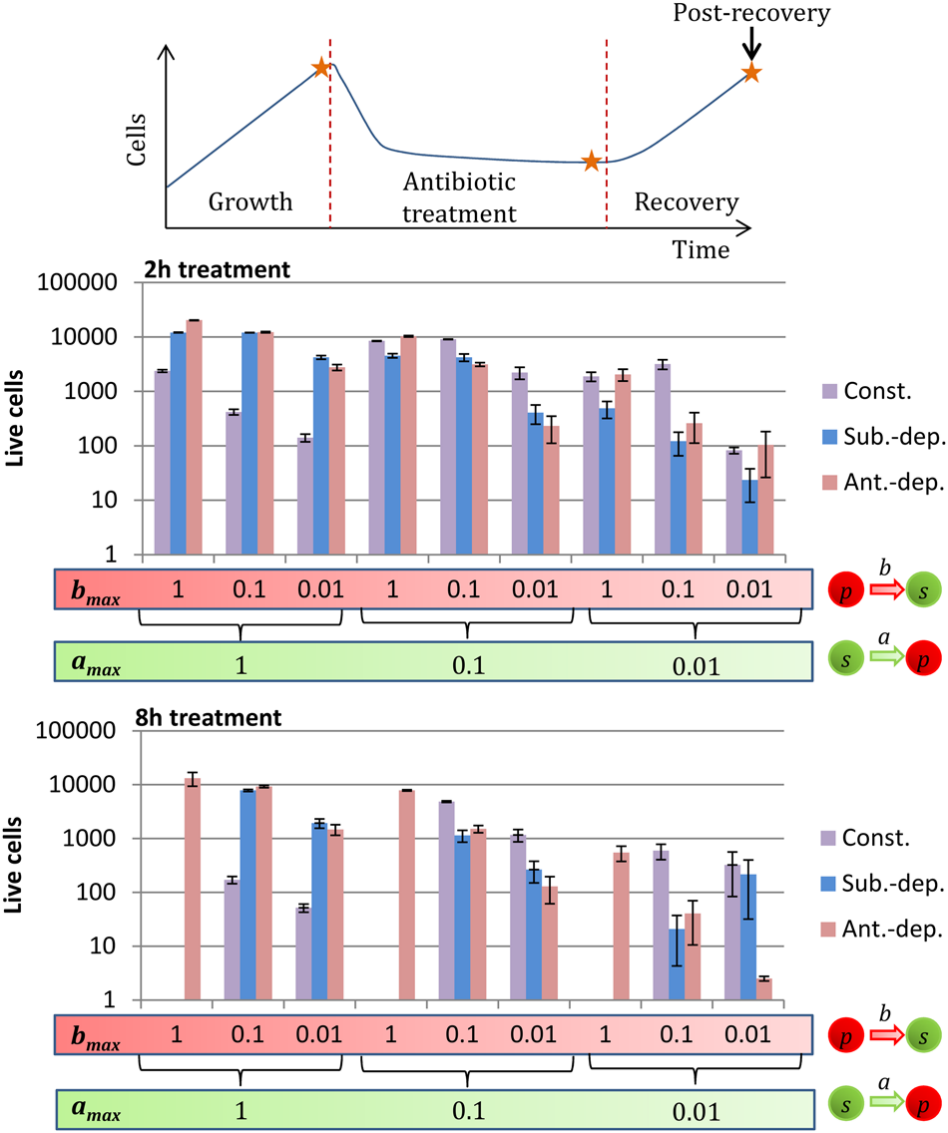
Quantity of live cells (susceptible cells + persisters) recovered 5 h after the treatment for the three switching strategies: constant (Const.), substrate-dependent (Sub.-dep.) and antibiotic-dependent (Ant.-dep.). *a*_max_ is the maximum switching rate from the susceptible state to the non-growing persister state. *b*_max_ is the maximum switching rate from the persister state to the susceptible state

After the antibiotic treatments (2 or 8 h long), the virtual biofilms were allowed to regenerate for 5 h. To achieve fast recovery, a great number of persisters had to switch back to the actively growing susceptible state immediately after treatment. This event depends on the number of persisters and on their switching rate post-treatment (Supplementary Figure S2). As stated previously, for constant and substrate-dependent switches, low wake-up rates favor survival. However, it also limits recovery. Thus, a compromise was needed between persister switches after and during treatment to achieve a high recovery. That is, low *b*_max_ values favor survival during treatments (Fig. 3) whereas high *b*_max_ values favor recovery after treatments. Consequently, the intermediate *b*_max_ value (0.1) led to the best recovery for these two strategies (Fig. 4). With antibiotic-dependent switches, persister switches were inhibited during treatment and induced by the removal of the antibiotic. Therefore, high *b*_max_ values did not lead to any enhanced mortality during treatment and allowed a quick recovery after treatment. On the other hand, a slow wake-up rate (i.e., low *b*_max_ values) allowed the pool of persisters formed during treatment to be maintained for a long time after the stress. Details regarding the population dynamics with the different strategies and parameter sets are available in the supplementary information of this article (Supplementary figures S3 to S8).

Altogether, the results showed that an increase in the duration of the treatments limited the recovery of the biofilms for constant and substrate-dependent strategies. In all cases, limiting growth by lowering substrate concentrations could be used to limit the recovery speed of the biofilms. However, for the substrate-dependent strategy, substrate depletion induced persister formation. With this strategy, the presence of substrate increased the efficacy of antibiotic treatments. This increase was not greatly effective with low *b*_max_ values. For the antibiotic-dependent strategy, the wake-up of persisters was triggered by the absence of antibiotic. As previously suggested by Cogan et al. (2013),^22^ periodic treatments may be effective by inducing persister switches into the susceptible state in the intervals between treatments. That being said, it is difficult to identify an optimal treatment strategy since its efficacy would largely depend on the switching strategy and its parameter set.

### Post-recovery structural patterns of biofilms depend on the spatial distribution of persisters

**Fig. 5:**
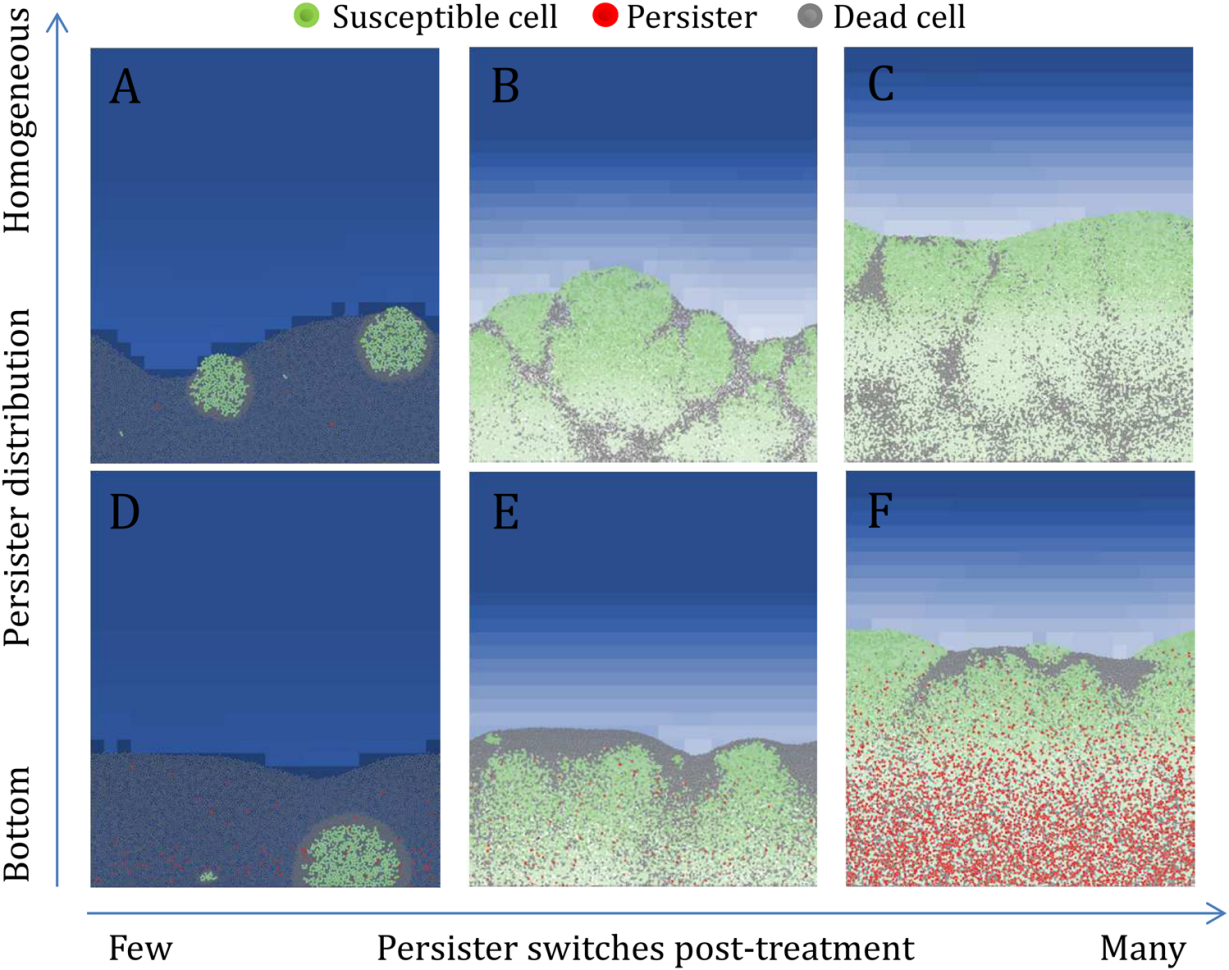
Structural patterns of the recovered biofilms 5 h after the treatment. The efficiency of the recovery depends on the number of persisters that have been able to switch to actively growing cells at the end of the treatment. This event depends on the number of persisters able to survive the treatment and on their switching rate *b*. A high rate of persister switches post-treatment leads to a fast recovery and thick biofilms after the 5 h of recovery. With the substrate-dependent strategy, persisters are mainly formed in substrate-deprived zones at the bottom of the biofilms. When persisters wake up at the bottom of the biofilms, the resulting colonies push against dead cells above them and form a shell of dead cells above the biofilms (**d**, **e**, **f**). With the constant and antibiotic-dependent strategies, persisters are homogeneously distributed in the biofilms. When persisters wake up randomly in the biofilms, the colonies push against dead cells around them and two colonies that encounter each other form characteristic strips of dead cells (**a**, **b**, **c**)

As seen in the previous paragraph, recovery efficiency depended on the number of persisters able to switch back to the growing susceptible state after treatment. However, the structures of the resulting biofilms were different, even with similar recovery rates, and depended on the spatial position of the persisters. Figure 5 shows biofilms with identical recovery time (5 h) but with different structures. When a few persisters woke up after treatment, only a few colonies were formed and the majority of the biofilms was composed of dead cells after the 5 h recovery period (Fig. 5a, d). If many persisters woke up post-treatment, the 5 h of recovery were sufficient to obtain thick biofilms (Fig. 5c, f). With a homogeneous distribution of persisters, colonies formed post-treatment pushed against the dead cells around them and colonies encountering each other left characteristic strips of dead cells between them (Fig. 5b, c). If persisters were mainly distributed in the bottom of the biofilms, colonies formed post-treatment pushed against dead cells above them and created a shell of dead cells above the biofilms (Fig. 5e, f).

New-formed colonies near to the top of the biofilms were closer to the substrate source and were thus at an advantage compared to the colonies at the bottom. Although the spatial distribution of persisters did not affect the recovery efficiency of the biofilms in our simulations, it could be a competitive advantage in multi-species biofilms to form colonies in strategical spatial positions such as the top of the biofilms. With the substrate-dependent strategy, persisters were formed in substrate-depleted zones at the bottom of the biofilms. With the constant and antibiotic-dependent strategies, persisters were homogeneously distributed, thereby increasing the probability to form colonies near the biofilm surface. All the post-recovery biofilms for the different strategies and parameter sets are presented in the supplementary information of this article (Supplementary figures S9 to S14).

### Increasing the maximal growth rate disproportionally affects substrate-concentration gradients in biofilms and hence survival with substrate-dependent switches

A local sensitivity analysis showed that the maximal growth rate, *µ*_max_, was the parameter that affected the most the outputs of the simulations (Supplementary figure S15). Increasing the maximal growth rate by 20% increased the total cells pre-treatment by 60% with all switching strategies. However, it increased the fraction of survivors up to 320% for the substrate-dependent strategy (with the parameter set *a*_max_ = 1 and *b*_max_ = 0.1), while *µ*_max_ did not much affect the fraction of survivors with the constant and antibiotic-dependent strategies. This enhanced survivability was directly related to the formation of higher proportions of persisters pre-treatment. *µ*_max_ affected the growth rate of susceptible cells and their substrate consumption. With an increased *µ*_max_, cells grew and consumed substrate faster, leading to thicker biofilms but without any increase in the number of actively growing cells, owing to the limitation of substrate availability. As a result, the proportion of starving cells in the biofilms increased with *µ*_max_. Since persister formation was maximum in substrate-depleted zones with substrate-dependent switches, higher proportions of persisters were obtained pre-treatment and hence better biofilm survival.

## Discussion

Bacterial persistence is ubiquitous in many bacterial species able to form biofilm such as *Escherichia coli*,^16^ *Staphylococcus aureus*,^18^ *Pseudomonas aeruginosa*,^31^ and *Klebsiella pneumoniae*.^25^ Given the ephemeral nature of persisters, their heterogeneity and the diversity of their formation mechanisms, it is difficult to relate the dynamics of persister populations within biofilms to their capacity to grow, survive and recover from biocide shocks such as antibiotic treatments. The simulations unveiled a variety of possible population dynamics according to the switching strategy between susceptible and persister cells and the parameter set used.

The diversity of persister dynamics observed made it difficult to generalize a treatment strategy to eradicate biofilms. The presence of persisters allowed the survival and recovery of biofilms with most of the parameter sets used. Devising a treatment strategy depends on the switching strategy, but also on the maximum switching rates. For example, with substrate-dependent switches, substrate can be added with the antibiotic to enhance the efficacy of treatments but it would have a limited effect if the maximum switching rate from persister to susceptible is low and would on the contrary favor biofilm growth, concentration gradients and persister formation. Thus, treatment strategies must be adapted to the persistence mechanisms and dynamics. The use of molecules able to target persisters directly seems inevitable and this strategy has already been proven to improve treatment efficacy.^32–34^ Several methods can combat persister cells, either by inhibiting their formation, waking them up or by killing them directly.^35–37^ However, the appropriateness of these methods depends on the mechanisms of persister formation and persister type.

Environment-dependent switches, compared to constant switches, were more effective in achieving biofilm recovery (Supplementary figures S3 to S8). Antibiotic-dependent switches enable the synchronization of the wake-up of persisters when the antibiotic concentration becomes low. This strategy led to the best recovery of the biofilms in our simulations. However, persisters are tolerant to various stresses in addition to antibiotics such as low pH and heat.^17^ To form persisters only in response to a particular antibiotic could impair the capacity of a bacterial population to overcome other types of stresses.

The resistance and resilience of the composition and function of microbial communities subject to disturbance are recurrent themes in microbial ecology, in which persister population dynamics could play an important role.^38^ Interspecies differences in persister population dynamics could explain shifts in natural biofilm community composition after disturbances. The population dynamics observed in our simulations were diverse, depending on the switching strategy and parameter set used (Supplementary figures S3 to S8). Efficient recovery and strategic spatial positions such as the top of the biofilm, close to the nutrient source, could give a competitive advantage after removal of stress. During simulations of competition between two or three strategies, the bacterial populations using the antibiotic-dependent strategy outcompeted the others during the recovery phase (Supplementary figure S16). Colonies recovered on the top of biofilms may also detach easily and by subsequently colonizing other surfaces gain a fitness advantage.^39^ However, multi-species biofilms may also share protection mechanisms that add to the complexity of their response to disturbances.^40,41^

Phenotypic heterogeneity allows bacterial populations to adopt a bet-hedging strategy to increase their fitness in fluctuating environments.^42^ Via phenotypic switches, sub-populations can arise with phenotypes adapted to new environmental conditions. Bacterial persistence may be a particular instance of this phenomenon, in which the phenotypic variants are tolerant to antibiotics. The rates of phenotypic switches can evolve within a few generations of cells and could be complementary to permanent phenotypic changes.^43^ Individual-based models are well-designed to represent cell individuality and are useful to simulate the phenotypic and spatial heterogeneity of cell populations. Because of the difficulty in observing individual cells, these models are emerging as a valuable complement to current experiments.

## Methods

### Overview of the individual-based biofilm model

The model constructed derives from previously developed individual-based biofilm models.^29,44^ Cells grow, divide and push against one another to generate a biofilm. Dissolved substrate and antibiotic diffuse and react with the cells in a diffusion-reaction fashion. They diffuse from a bulk liquid above the biofilm, where concentrations are maintained constant, across a boundary layer toward the biofilm (Figure S1). In the model, bacteria can be susceptible, persister or dead. Unlike in a few previous models,^29,45^ detachment, cell maintenance, shrinking of the biofilm and EPS production are not taken into account.^26^ The simulated time is assumed to be low enough to limit the effect of these factors. The computational model is implemented in NetLogo. Although the computation time is long when there are many cells, the graphical interface of NetLogo makes the model easy to manage and to modify.

### Simulation process

At the start of a simulation, 10 susceptible bacteria are set up randomly on the surface (*y* = *0*). The initial concentration of substrate in all the computational domain is equal to the concentration in the bulk, *C*_S,bulk_. The diffusion-reaction dynamics and cell dynamics operate at different time scales and we assume that diffusion-reaction is at steady state when cells are updated.^45,46^ The default value of the time step of cell update (Δ*t*_cell_) is one minute. Results presented are the means of four simulations with different random seeds.

### Cell growth

Cell growth follows a Monod kinetic model. There is only one growth-limiting substrate that each susceptible cell *i* consumes to increase its mass *m*_*i*_. We assume that persister cells do not grow or consume substrate. *m*_*i*_ varies according to equation 1.^47,48^ *C*_*S*_ is the substrate concentration, *µ*_max_ is the maximal specific growth rate and *K*_*S*_ is the half-saturation constant for substrate *S*. Cells are cylinders of length Δ*l* and diameter *d*_*i*_ that depends on cell mass and density *ρ* (equation 2).

1 $$\frac{{{\boldsymbol{dm}}_{\boldsymbol{i}}}}{{{\boldsymbol{dt}}}} = {\boldsymbol{m}}_{\boldsymbol{i}}.{\mathrm{\mu }}_{{\boldsymbol{max}}}.\frac{{{\boldsymbol{C}}_{\boldsymbol{S}}}}{{{\boldsymbol{C}}_{\boldsymbol{S}} + {\boldsymbol{K}}_{\boldsymbol{S}}}}$$

2 $${\boldsymbol{d}}_{\boldsymbol{i}} = \sqrt {\frac{{4 \times {\boldsymbol{m}}_{\boldsymbol{i}}}}{{{\boldsymbol{\rho }} \times \Delta {\boldsymbol{l}} \times {\boldsymbol{\pi }}}}}$$

Cell division is triggered by a threshold mass *m*_max_.^29,49^ To avoid division synchronization, daughter cells randomly receive 40 to 60 percent of the mass of their mother cell from a uniform distribution.^50^ The sum of the mass of the two daughter cells is equal to that of their mother. Their centers are randomly set opposite each other on the edge of the mother cell, without overlapping.

### Shoving algorithm

Cellular growth and division may create overlaps between cells. These overlaps are relaxed by a shoving algorithm.^27,29,45^ If a bacterium overlaps one or more neighbors, it moves by a vector directed from the center of the overlapping neighbor to its own center. The shoving vector of a cell *i* (*u*_*S,i*_) is calculated by equation 3 with *a*_*i*_ the radius of the cell, *a*_*h*_ the radius of the neighbor *h*, *d*_*h*_ the distance between the center of the two overlapping cells and *u*_*h*_ a unitary vector directed from the center of neighbor *h* to the center of cell *i*. The shoving algorithm is run until less than five percent of the cells are still moving or if it reaches a maximum number of iterations. To avoid heavy computation time, this maximum is set at one thousand iterations per cell update.

3 $$u_{S,i} = \mathop {\sum }\limits_{h = 1}^N \frac{{a + a_h - d_h}}{2}.u_h$$

### Antibiotic treatment

At the start of the antibiotic treatments, the antibiotic concentration in the bulk is set to *C*_A,bulk_ and the antibiotic diffuses toward the biofilm. At the end of the antibiotic treatments, the antibiotic is set to zero in the bulk and leaves the biofilm by diffusion. High concentrations of antibiotics are used so that they quickly diffuse into the biofilms and kill susceptible cells. If the antibiotic concentration *C*_A_ is greater than the minimum inhibitory concentration (MIC), the killing rate *k* of the susceptible population is determined by equation 4. The killing rate of the persister population *k*_*p*_ is determined by equation 5. *k*_max_ is the maximum killing rate of susceptible cells and *k*_maxp_ is the maximum killing rate of persisters. *K*_*A*_ is a constant so that *k(C*_*A*_ = *MIC)* = *µ*_max_. Hence, when *C*_*A*_ ≥ *MIC*, the growth rate of the population cannot exceeds its killing rate, which would contradict the definition of the MIC.

4 $${\boldsymbol{k}}({\boldsymbol{C}}_{\boldsymbol{A}}) = {\boldsymbol{k}}_{{\boldsymbol{max}}}.\frac{{{\boldsymbol{C}}_{\boldsymbol{A}}}}{{{\boldsymbol{C}}_{\boldsymbol{A}} + {\boldsymbol{K}}_{\boldsymbol{A}}}}$$

5 $${\boldsymbol{k}}_{\boldsymbol{p}}({\boldsymbol{C}}_{\boldsymbol{A}}) = {\boldsymbol{k}}_{{\boldsymbol{maxp}}}.\frac{{{\boldsymbol{C}}_{\boldsymbol{A}}}}{{{\boldsymbol{C}}_{\boldsymbol{A}} + {\boldsymbol{K}}_{\boldsymbol{A}}}}$$

Persisters have a better survivability than susceptible cells because *k*_max_ » *k*_maxp_. To convert these killing rates into individual probabilities of dying, at each time step Δ*t*_cell_, a random number between 0 and 1 is produced. If the number is below Δ*t*_cell_×*k*(*C*_A_) or Δ*t*_cell_×*k*_*p*_(*C*_*A*_), the cell dies. The cells have different probabilities of dying depending on their phenotype and local antibiotic concentration. Dead cells become inactive and remain in the computational domain for the remainder of the simulation.^51^ They continue to participate in the shoving algorithm but their diameter is reduced by 20% compared to growing cells and persisters to take into account their shrinking. There is little feedback from previous models on the fate of dead cells in treated biofilms.^51,52^ We chose to have the treated biofilms keep their structure intact despite being dead.^53^ Lardon et al. (2011) hypothesized that dead cells progressively convert their mass into substrate and this approach was used in a few planktonic batch models,^29,54,55^ In our model, dead cells are lysed at a rate *L*_*DS*_. If their size goes under a minimal threshold, they are removed, the effect of this process on mass conservation being small.

### Diffusion-reaction of solutes

The solutes, namely the substrate and the antibiotic, diffuse in the environment. The general mass balance for a component *n* of concentration *C*_*n*_ is set up by a partial differential equation (equation 6).

6 $$\frac{{{\boldsymbol{dC}}_{\boldsymbol{n}}}}{{{\boldsymbol{dt}}}} = {\boldsymbol{D}}_{\boldsymbol{n}}.\nabla .{\boldsymbol{C}}_{\boldsymbol{n}} + {\boldsymbol{r}}_{\boldsymbol{n}}$$

$\nabla = \overrightarrow {\boldsymbol{i}} \frac{{\boldsymbol{d}}}{{{\boldsymbol{dx}}}} + \overrightarrow {\boldsymbol{j}} \frac{{\boldsymbol{d}}}{{{\boldsymbol{dy}}}}$ is the vector gradient operator. *x* and *y* are spatial coordinates. *D*_*n*_ is the diffusion coefficient of component *n*. *r*_*n*_ is the consumption (negative) or production (positive) of component *n*. This equation is resolved by discretizing the computational space.^29,44,56^ Diffusion-reaction occurs on a grid with voxels of length Δ*l* (see Supplementary Information). We assumed the diffusion inside the biofilm is constant although limited. The diffusion coefficient inside the biofilm is *D*^*b*^_*n*_. We set *D*^*b*^_*n*_ = 0.8*×D*_*n*_.^29^ A few antibiotics such as ciprofloxacin have been reported to diffuse quickly in biofilms and we assumed that limited penetration does not protect the biofilms.^57^ In addition, we used high antibiotic doses in our simulations. Lateral boundaries of the computational domain are periodic. Cells and solutes cannot penetrate the surface under the biofilm. Concentrations are kept constant in the bulk. The consumption of substrate on a grid cell is the sum of the uptake of each bacterium in it.^47^ It is defined by equation 7. *X*_*x,y*_ is the biomass of growing cells on the grid cell located at (*x;y)* and *Y*_*XS*_ is the biomass yield. *X*^*d*^_*x,y*_ is the mass of dead cells on the grid cell located at (*x;y)* and *L*_DS_ is the lysis rate of dead biomass. We assumed that antibiotic consumption is zero (*r*_*A*_ = 0).

7 $$r_S = - X_{x,y} \times {\mathbf{\mu }}_{{\boldsymbol{max}}}.\frac{{{\boldsymbol{C}}_{\boldsymbol{S}}}}{{{\boldsymbol{C}}_{\boldsymbol{S}} + {\boldsymbol{K}}_{\boldsymbol{S}}}} \times \frac{1}{{{\it{Y}}_{{\boldsymbol{XS}}}}} + X_{x,y}^d \times L_{DS}$$

### Phenotypic switches between susceptible and persister cells

We derived three switching strategies from the switching models of Carvalho et al.^25^ The switching models are defined by the equations 8 to 13 where *a*_max_, *b*_max_, *K* and *K’* are constants. For the default parameters, we chose *K* = *K*_*S*_ and *K’* = MIC. *a(C*_*S*_*)* is maximum when *C*_*S*_ « *K* and close to zero when *C*_*S*_ » *K*, inversely for *b*(*C*_*S*_). *a*(*C*_*A*_) is maximum when *C*_*A*_ » *K’* and close to zero when *C*_*A*_ « *K’*, inversely for *b(C*_*A*_*)*. The switching rates *a* and *b* are converted to individual switching probabilities for simulations.

Strategy constant switches:

8 $${\boldsymbol{a}} = {\boldsymbol{a}}_{{\boldsymbol{max}}}$$

9 $${\boldsymbol{b}} = {\boldsymbol{b}}_{{\boldsymbol{max}}}$$

Strategy substrate-dependent switches:

10 $${\boldsymbol{a}}({\boldsymbol{C}}_{\boldsymbol{S}}) = {\boldsymbol{a}}_{{\boldsymbol{max}}} \times \left( {1 - \frac{{{\boldsymbol{C}}_{\boldsymbol{S}}}}{{{\boldsymbol{C}}_{\boldsymbol{S}} + {\boldsymbol{K}}}}} \right)$$

11 $${\boldsymbol{b}}({\boldsymbol{C}}_{\boldsymbol{S}}) = {\boldsymbol{b}}_{{\boldsymbol{max}}} \times \left( {\frac{{{\boldsymbol{C}}_{\boldsymbol{S}}}}{{{\boldsymbol{C}}_{\boldsymbol{S}} + {\boldsymbol{K}}}}} \right)$$

Strategy antibiotic-dependent switches:

12 $${\boldsymbol{a}}({\boldsymbol{C}}_{\boldsymbol{A}}) = {\boldsymbol{a}}_{{\boldsymbol{max}}} \times \left( {\frac{{{\boldsymbol{C}}_{\boldsymbol{A}}}}{{{\boldsymbol{C}}_{\boldsymbol{A}} + {\boldsymbol{K{\prime}}}}}} \right)$$

13 $${\boldsymbol{b}}({\boldsymbol{C}}_{\boldsymbol{A}}) = {\boldsymbol{b}}_{{\boldsymbol{max}}} \times \left( {1 - \frac{{{\boldsymbol{C}}_{\boldsymbol{A}}}}{{{\boldsymbol{C}}_{\boldsymbol{A}} + {\boldsymbol{K{\prime}}}}}} \right)$$

### Local sensibility analysis

The default parameters used for the simulations are presented in supplementary table S1. To analyze the sensibility of the model to the different parameters, we separately decreased or increased each parameter by twenty percent and compared total live cells pre-treatment, post-treatment and post-recovery with the default results (Supplementary figure S15). For each strategy, the switching rate parameters used were those that gave the most live cells after recovery with the default parameters: *a*_max_ = 0.1 and *b*_max_ = 0.1 for constant switches, *a*_max_ = 1 and *b*_max_ = 0.1 for substrate-dependent switches, *a*_max_ = 1 and *b*_max_ = 1 for antibiotic-dependent switches.

### Code availability

The source code of the computational model used is available on the NetLogo website (https://ccl.northwestern.edu/netlogo/) under the name ‘Bacterial persistence in biofilms’ or by requesting the authors.

### Data availability

All data relevant to the article is included in the article and its supplementary information.

## Electronic supplementary material

Supplementary Information
