# Supplementary material for: How do environment-dependent switching rates between susceptible and persister cells affect the dynamics of biofilms faced with antibiotics?
Source: NPJ Biofilms Microbiomes. 2018 Mar 15;4:6. doi: 10.1038/s41522-018-0049-2 (PMC5854711; doi:10.1038/s41522-018-0049-2)
Supplement: Supplementary file 1 — Supplementary Information [file 41522_2018_49_MOESM1_ESM.pdf]

How do environment-dependent switching rates between susceptible and persister cells affect the dynamics of biofilms faced with antibiotics?

Gabriel Carvalho[1*], Damien Balestrino[2], Christiane Forestier[2], Jean-Denis Mathias[1]

[1]UR LISC Laboratoire d'Ingénierie pour les Systèmes Complexes, Irstea, Aubière, France

[2]LMGE, UMR6023 CNRS, Université Clermont Auvergne, Clermont-Ferrand, France

***Correspondence:** IRSTEA, Laboratoire d'Ingénierie pour les Systèmes Complexes, 9 avenue Blaise Pascal, CS 20085, 63178 Aubière, France
jean-denis.mathias@irstea.fr; tel: +33 4 73 44 06 80; fax: +33 4 73 44 06 96

1. Computational domain

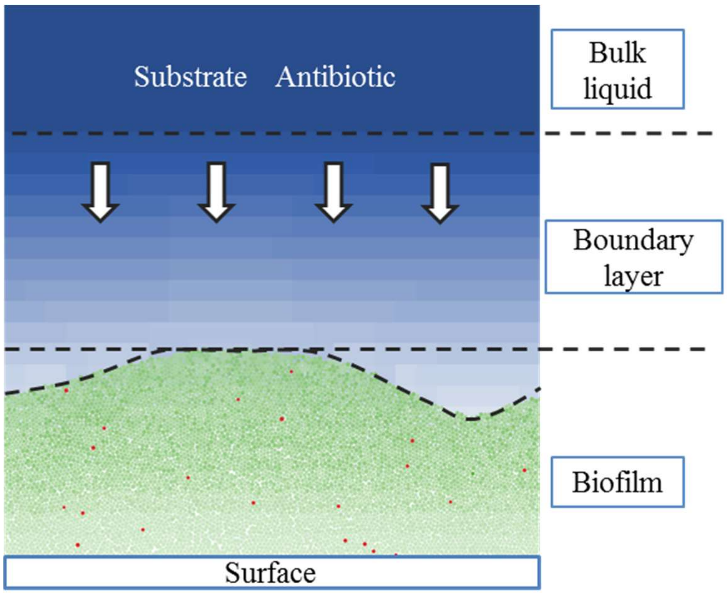

Figure S 1: Scheme of the spatial organization of the computational domain. The model is in two spatial dimensions, width and height. Solutes diffuse from a bulk liquid above the biofilm through a boundary layer. Actively growing susceptible cells can become non-growing persisters or dead cells. Persister can switch back to susceptible cells or become dead. Substrate concentration is represented by a shade of blue. Susceptible cells are represented by a shade of green, depending on their growth rate.

2. Model parameters

Table S1: Model default parameters.

Symbol	Units	Value	Description	Reference
**Computational domain**				
lx	µm	104	Width of the domain	This study
Δl	µm	4	Length of a grid cell (square)	This study
lb	µm	40	Length of the boundary layer	[1]
Δt_{dif}	ms	3.5	Time step of the diffusion-reaction algorithm	This study
Δt_{cell}	s	60	Time step of cell update	This study
**Solutes**				
$C_{S,bulk}$	$g.L^{-1}$	0.4	Substrate concentration in the bulk	This study
D_S	$µm^2.s^{-1}$	900	Diffusion coefficient of the substrate (glucose) in water at 37°C	[2]
$C_{A,bulk}$	xMIC	1000	Bulk concentration of antibiotic during treatments	This study
$C_{A,MIC}$	$mg.L^{-1}$	0.05	Minimal Inhibitory Concentration of the antibiotic	[3]
D_A	$µm^2.s^{-1}$	900	Diffusion coefficient of the antibiotic in water at 37°C	This study
D^b_n/D_n		0.8	Relative diffusion coefficient of a solute *n* (substrate *S* or antibiotic *A*) in the biofilm compared to its coefficient in water (D^b_S=0.8*D_S and D^b_A=0.8*D_A)	[1]
**Bacterial growth**				
μ_{max}	h^{-1}	1.25	Maximal growth rate	[3]
K_s	$g.L^{-1}$	$3.5x10^{-5}$	Half-saturation constant for the substrate *S* ($\mu=\mu_{max}/2$ for $C_S=K_s$)	[3]
Y_{XS}	$fg.cell^{-1}$	1187	Substrate consumed to produce one cell	[3]
d_X	$fg.µm^{-3}$	200	Density of cells	[4]
m_{div}	fg	500	Cell mass at division	[4]
k_{max}	h^{-1}	10	Maximum killing rate of normal cells	This study
k_{maxp}	h^{-1}	0.1	Maximum killing rate of persisters	This study
K_k	$g.L^{-1}$	6.4	Half-saturation constant for the killing rates of susceptible and persister cells ($k(MIC)=\mu_{max}$)	This study
L_{DS}	h^{-1}	0.05	Lysis rate of dead cells	This study

3. Diffusion-reaction algorithm

To be solved, the diffusion-reaction equation is discretized in time and space. The equation used in the model is:

$$C_n^{t+\Delta t_{diff}}(x,y) = C_n^t(x,y) + \frac{\Delta t_{diff}}{\Delta l^2}\begin{bmatrix} D_n(x-1,y) \times C_n^t(x-1;y) + D_n(x+1,y) \times C_n^t(x+1;y) \\ +D_n(x,y-1) \times C_n^t(x;y-1) + D_n(x,y+1) \times C_n^t(x;y+1) - N \times D_n(x,y) \times C_n^t(x;y) \end{bmatrix} + r_n(x,y) \times \Delta t_{diff}$$

Δt_{diff} is the time step. The space is discretized in square shaped grid cells of length Δl. $C_n^t(x,y)$ is the concentration of the component n in the patch (x, y) at an instant t. N is the number of neighbors of the grid cell (x, y). $D_n(x,y)$ is the diffusion coefficient of the component n in the grid cell (x,y). $r_n(x,y)$ is the set of reactions occurring in the grid cell (x, y) for the component n.

4. Relation between the number of persisters and *b* post-treatment and the recovery efficiency

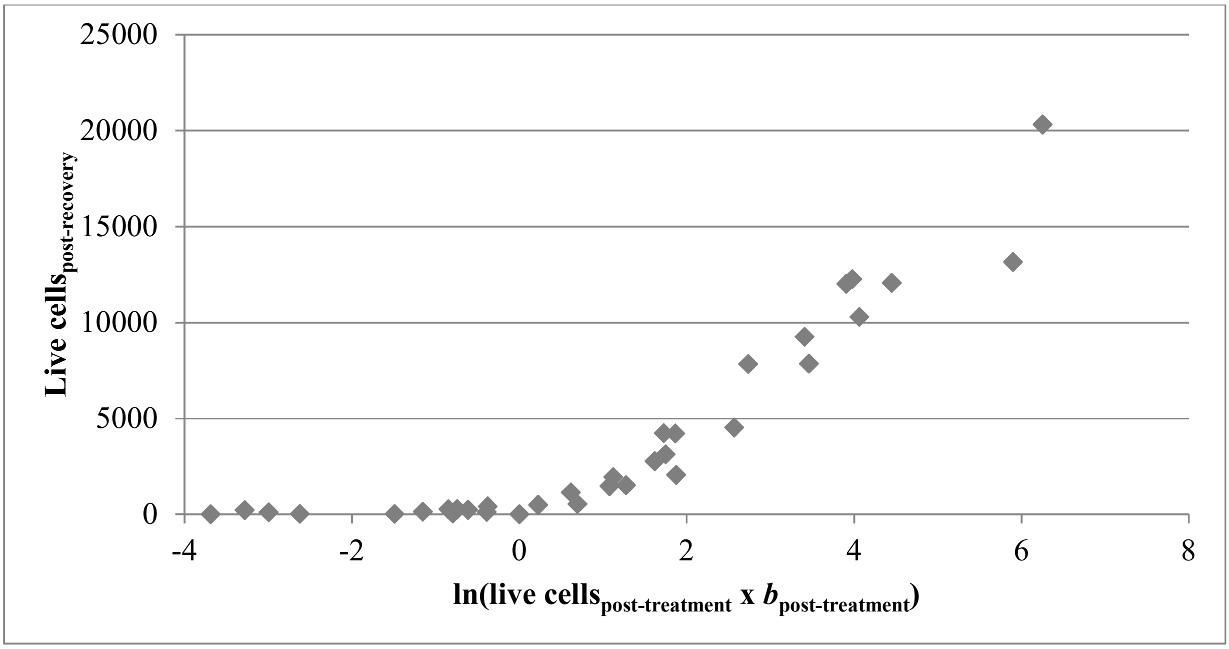

Figure S2: Correlations. Live cells post-recovery depends on the number of live cells post-treatment (only persisters) and the post-treatment wake up rate b when the switching strategy does not impair growth (substrate-dependent and antibiotic-dependent strategies). The x axis represents the natural logarithm of live cells post-treatment (i.e. the persisters which survived) multiplied by b_{max}. The results plotted are the mean of four simulations with the substrate or antibiotic-dependent strategies.

5. Population dynamics

5.1 Constant switches

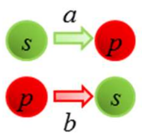

Set n°	1	2	3	4	5	6	7	8	9
a_{max} (h^{-1})	1	1	1	0.1	0.1	0.1	0.01	0.01	0.01
b_{max} (h^{-1})	1	0.1	0.01	1	0.1	0.01	1	0.1	0.01

5.1.1 2 hours treatment

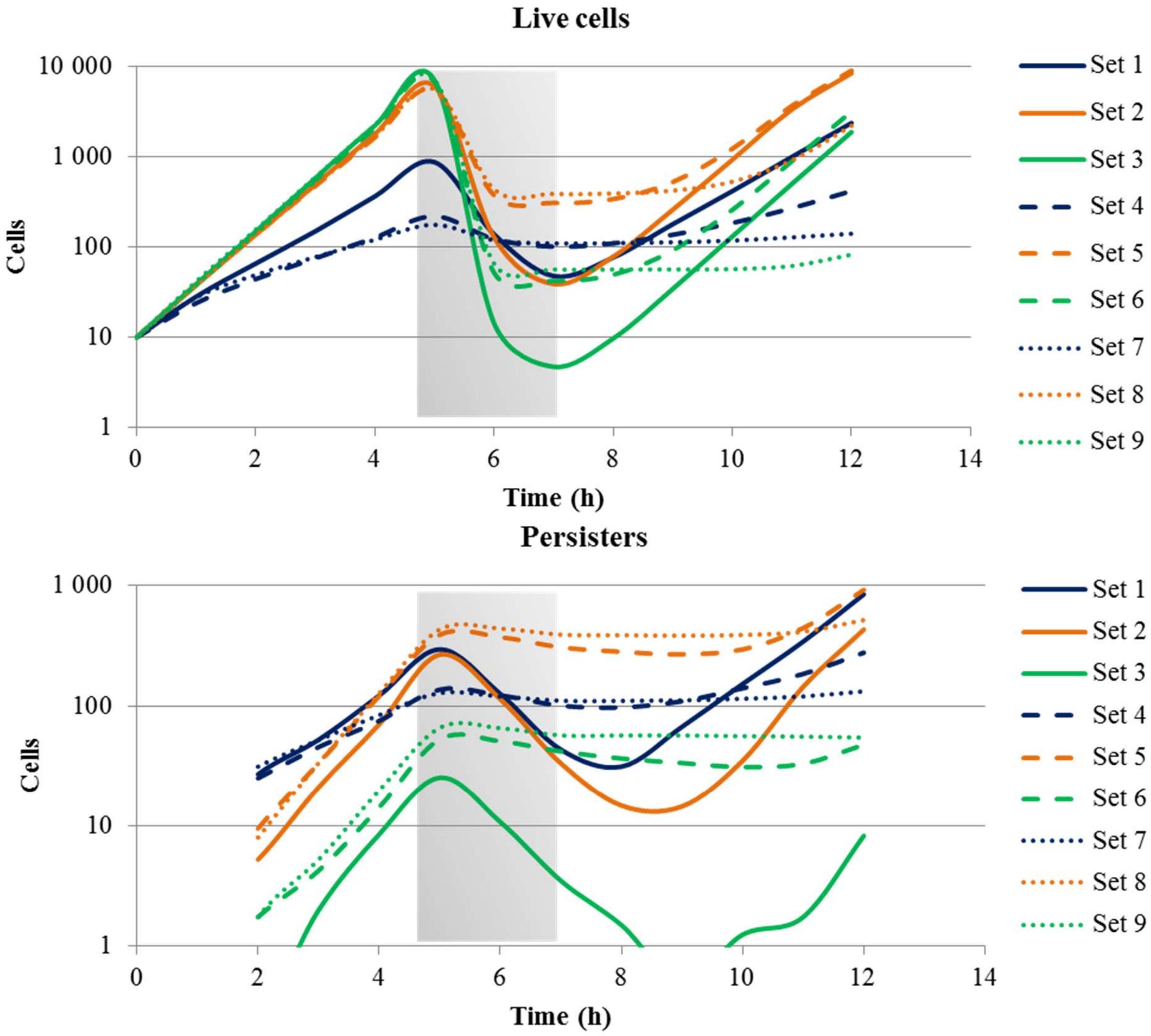

Figure S 3: Population dynamics of live cells (susceptible and persister cells) and persister cells only with constant switches, two hours treatment (means of four simulations). The shaded grey area represents the antibiotic treatment.

5.1.2 8 hours treatment

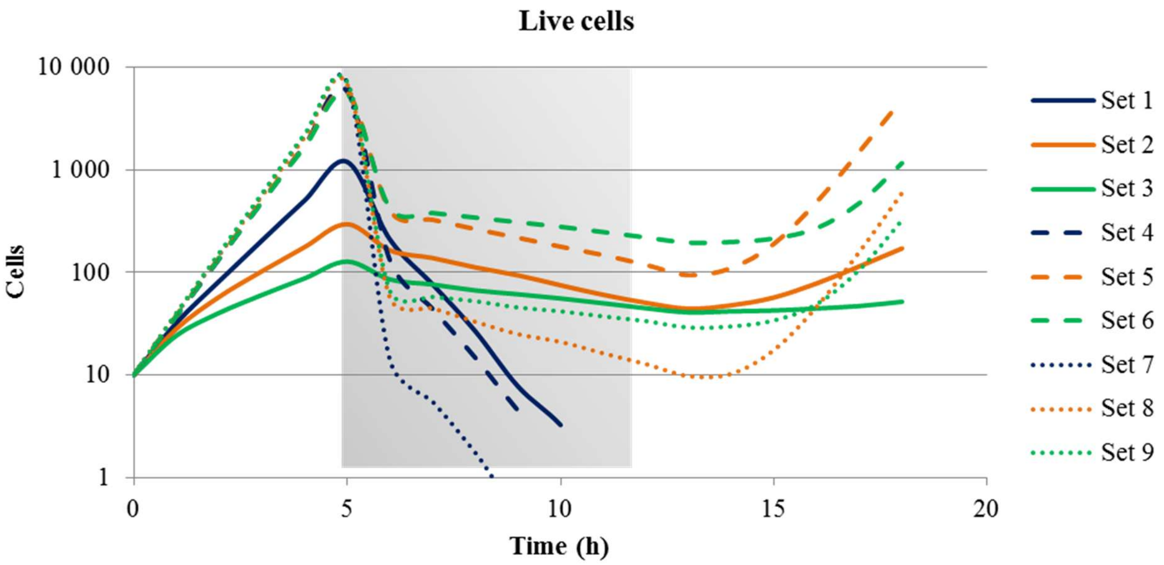

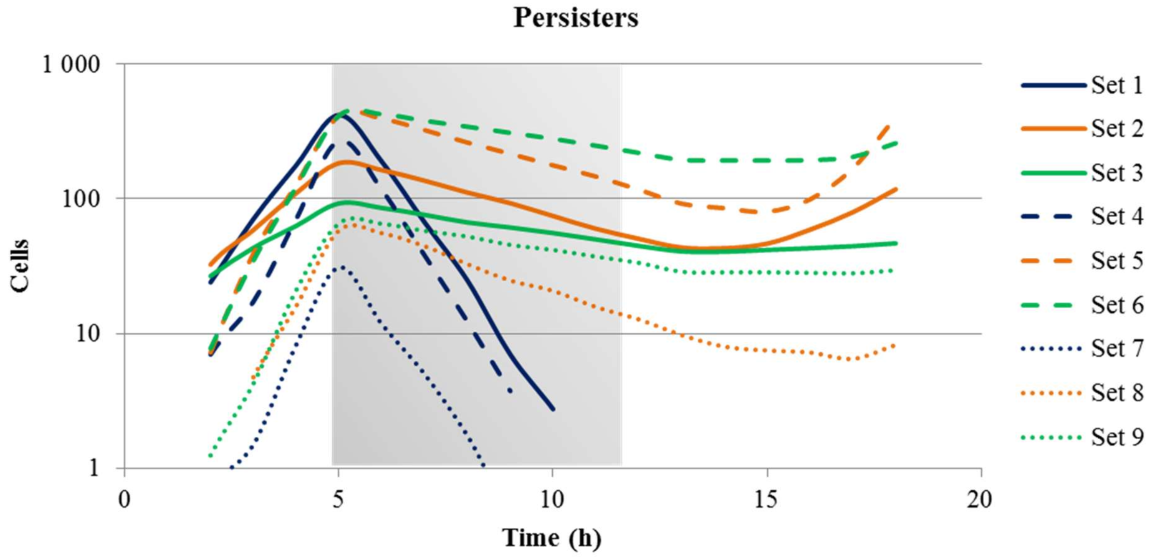

Figure S 4: Population dynamics of live cells (susceptible and persister cells) and persister cells only with constant switches, eight hours treatment (means of four simulations). The shaded grey area represents the antibiotic treatment.

5.2 Substrate-dependent switches

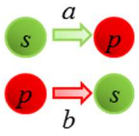

Set n°	1	2	3	4	5	6	7	8	9
a_{max} (h^{-1})	1	1	1	0.1	0.1	0.1	0.01	0.01	0.01
b_{max} (h^{-1})	1	0.1	0.01	1	0.1	0.01	1	0.1	0.01

5.2.1 2 hours treatment

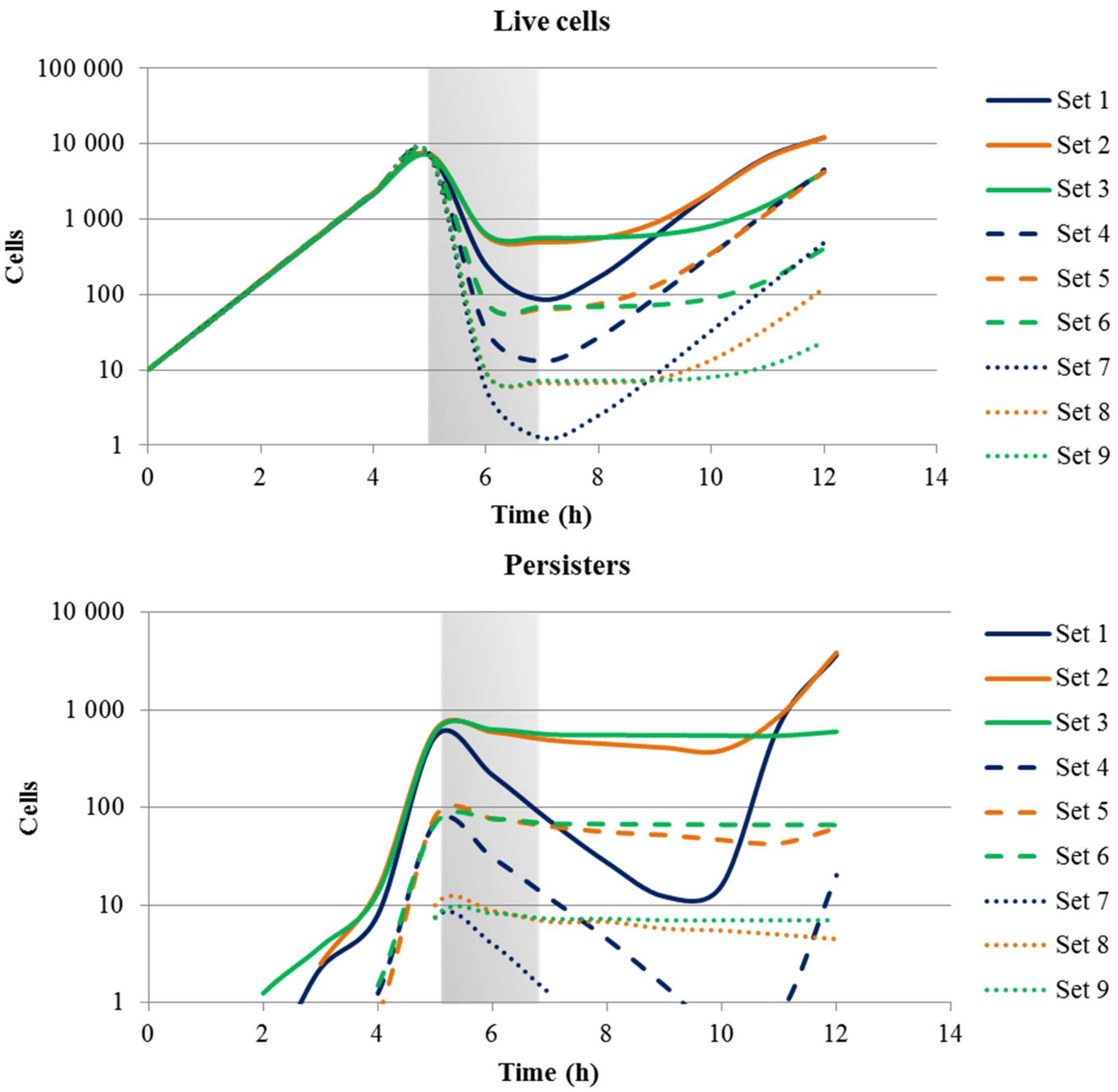

Figure S 5: Population dynamics of live cells (susceptible and persister cells) and persister cells only with substrate-dependent switches, two hours treatment (means of four simulations). The shaded grey area represents the antibiotic treatment.

5.2.2 8 hours treatment

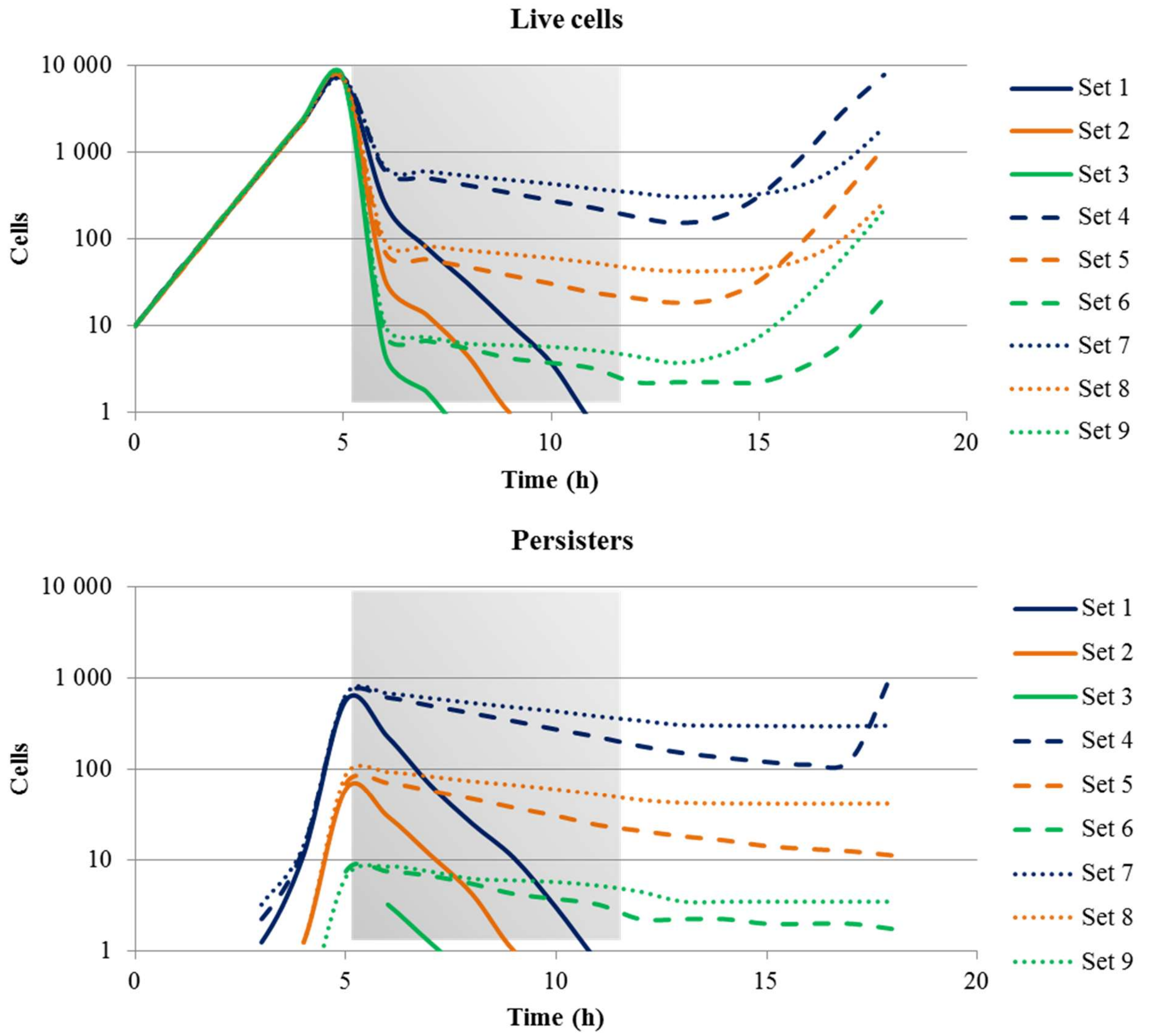

Figure S 6: Population dynamics of live cells (susceptible and persister cells) and persister cells only with substrate-dependent switches, eight hours treatment (means of four simulations). The shaded grey area represents the antibiotic treatment.

5.3 Antibiotic-dependent switches

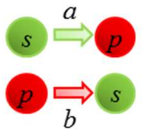

Set n°	1	2	3	4	5	6	7	8	9
a_{max} (h^{-1})	1	1	1	0.1	0.1	0.1	0.01	0.01	0.01
b_{max} (h^{-1})	1	0.1	0.01	1	0.1	0.01	1	0.1	0.01

5.3.1 2 hours treatment

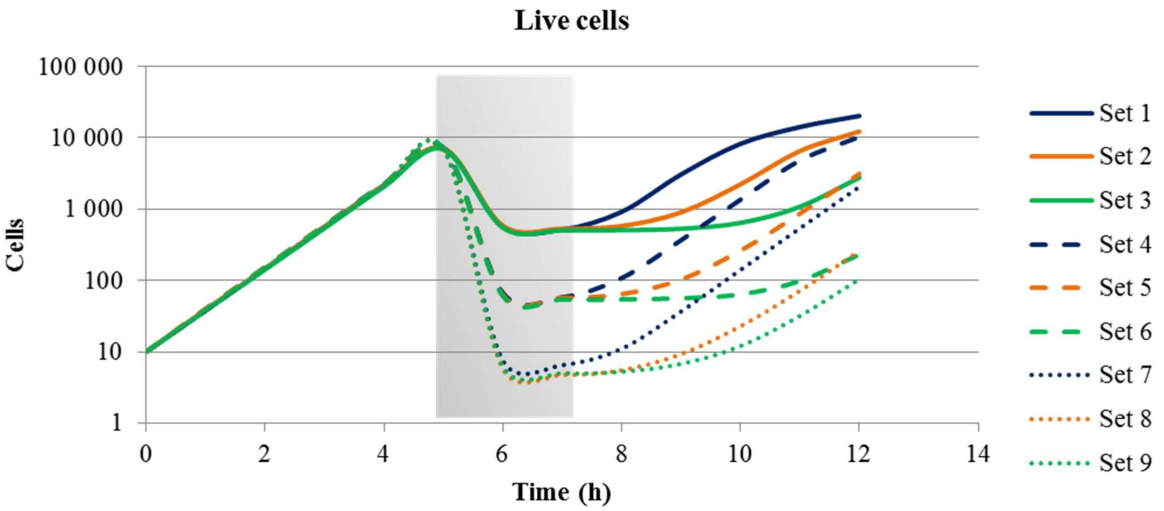

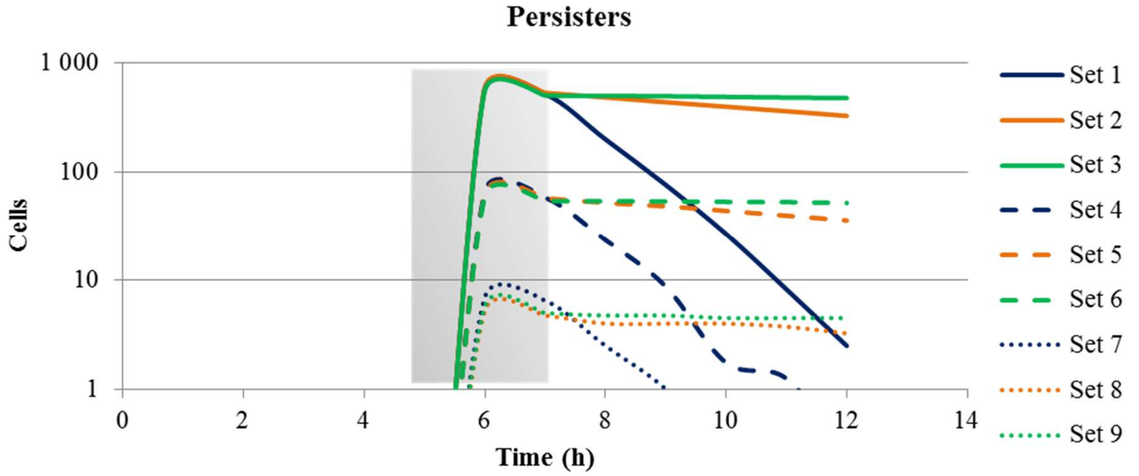

Figure S 7: Population dynamics of live cells (susceptible and persister cells) and persister cells only with antibiotic-dependent switches, two hours treatment (means of four simulations). The shaded grey area represents the antibiotic treatment.

5.3.2 8 hours treatment

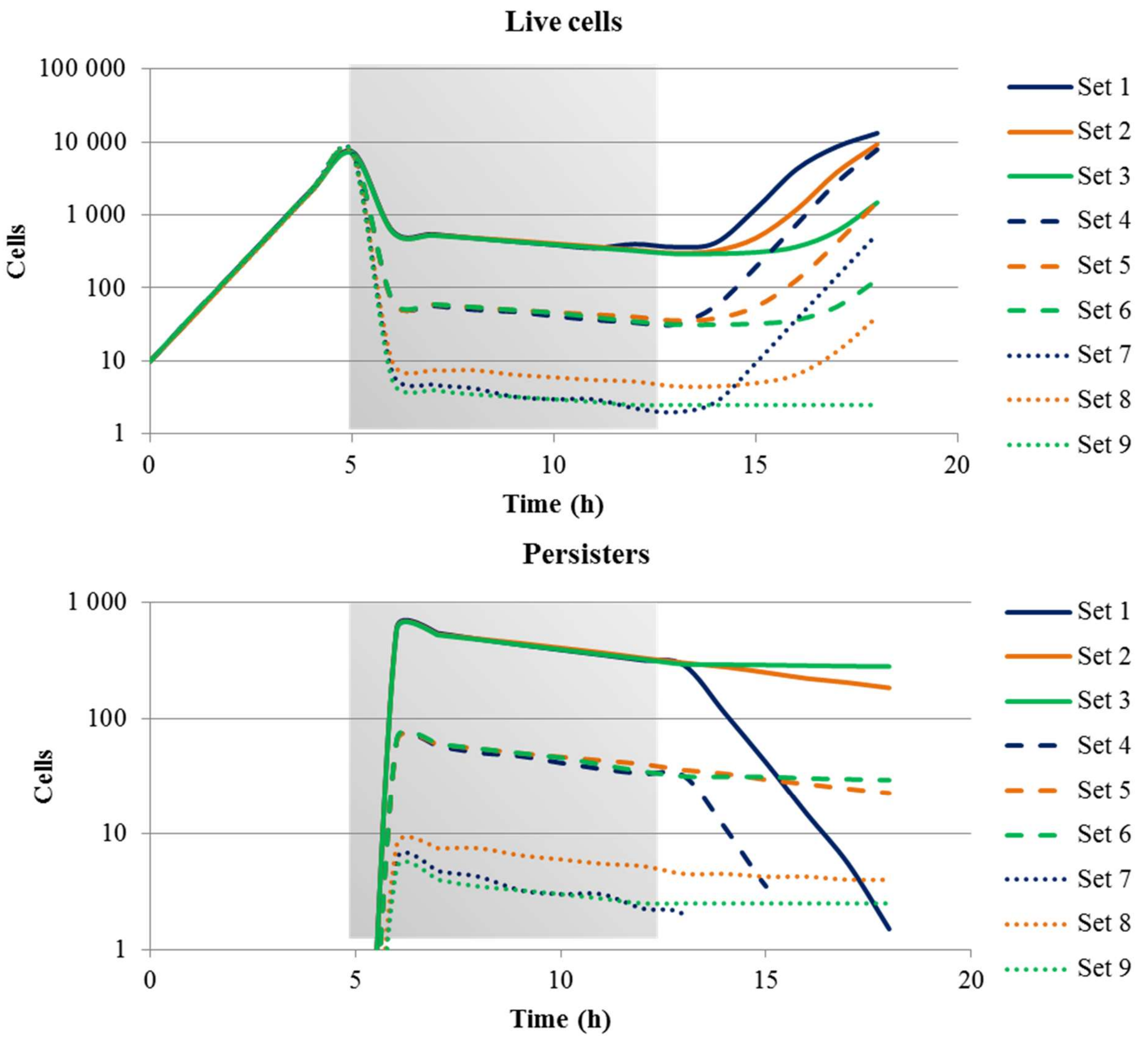

Figure S 8: Population dynamics of live cells (susceptible and persister cells) and persister cells only with antibiotic-dependent switches, eight hours treatment (means of four simulations). The shaded grey area represents the antibiotic treatment.

6. Images of recovered biofilms (5 hours post treatment)

6.1 Constant switches

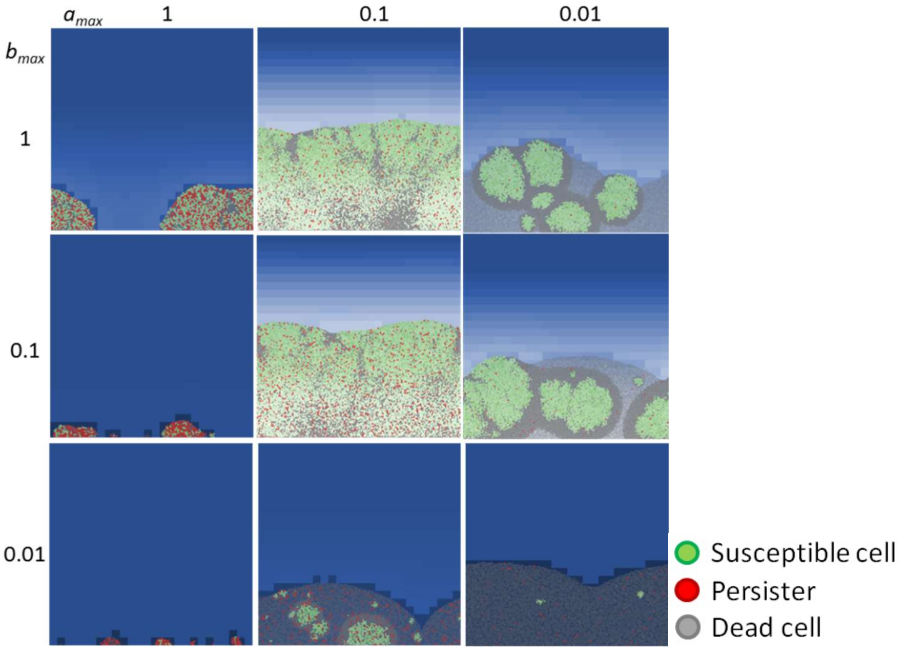

Figure S 9: Biofilms initially treated during two hours, constant switches.

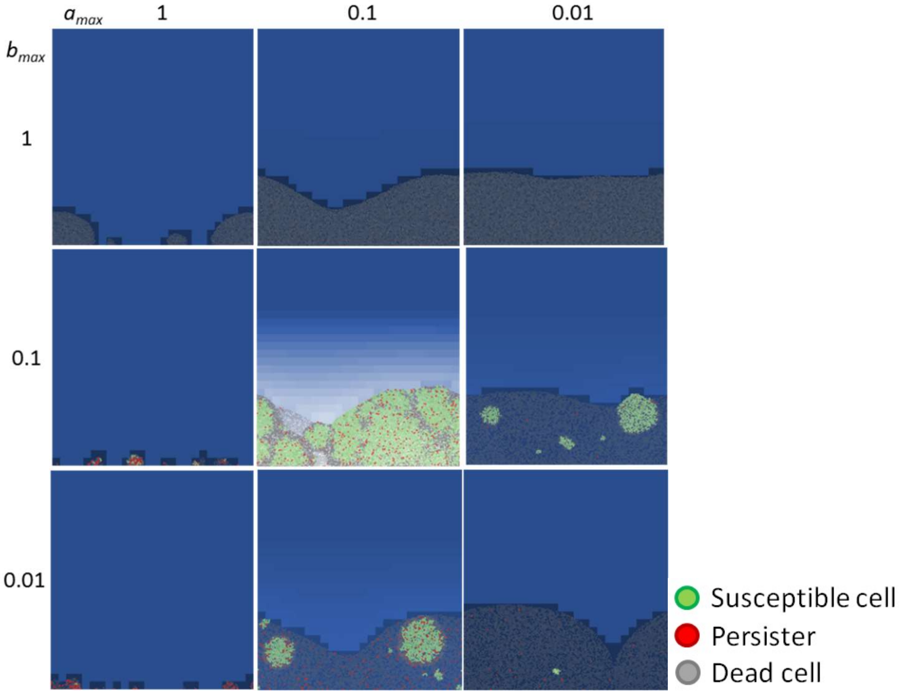

Figure S 10: Biofilms initially treated during eight hours, constant switches.

6.2 Substrate-dependent switches

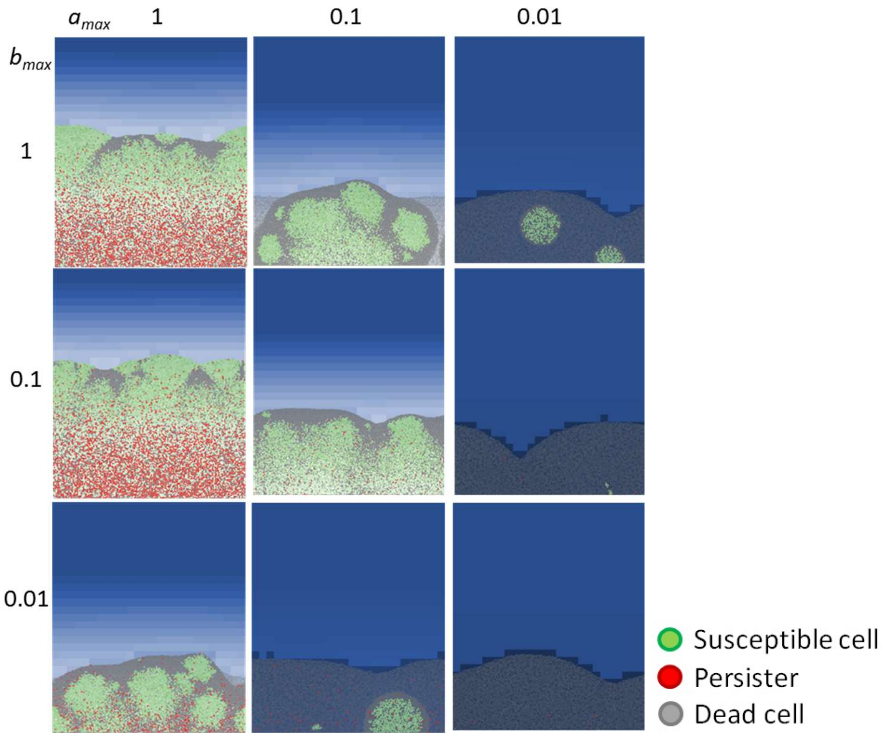

Figure S 11: Biofilms initially treated during two hours, substrate-dependent switches.

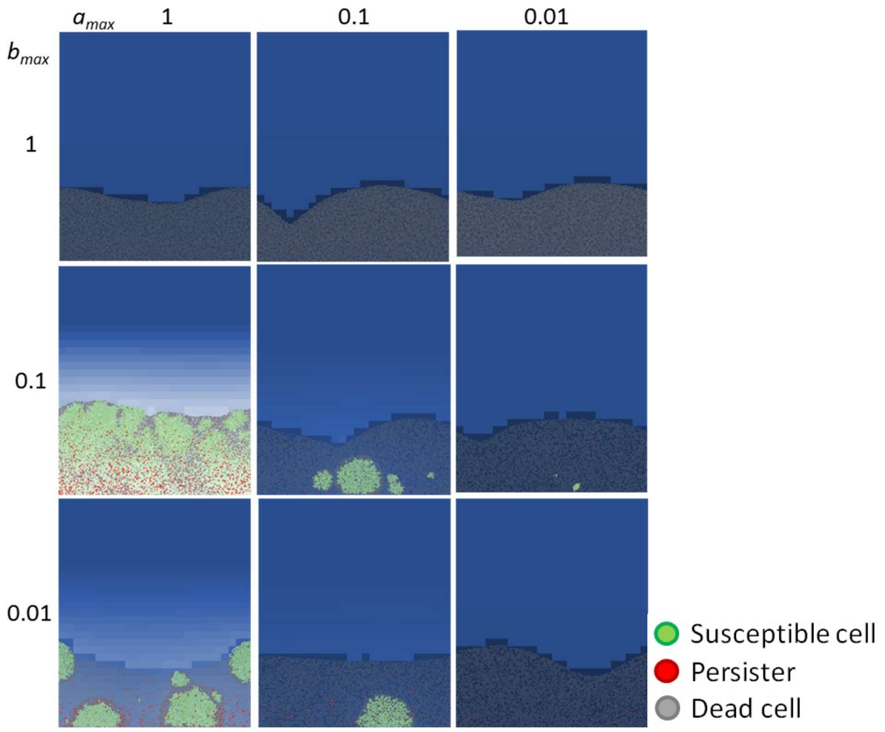

Figure S 12: Biofilms initially treated during eight hours, substrate-dependent switches.

6.3 Antibiotic-dependent switches

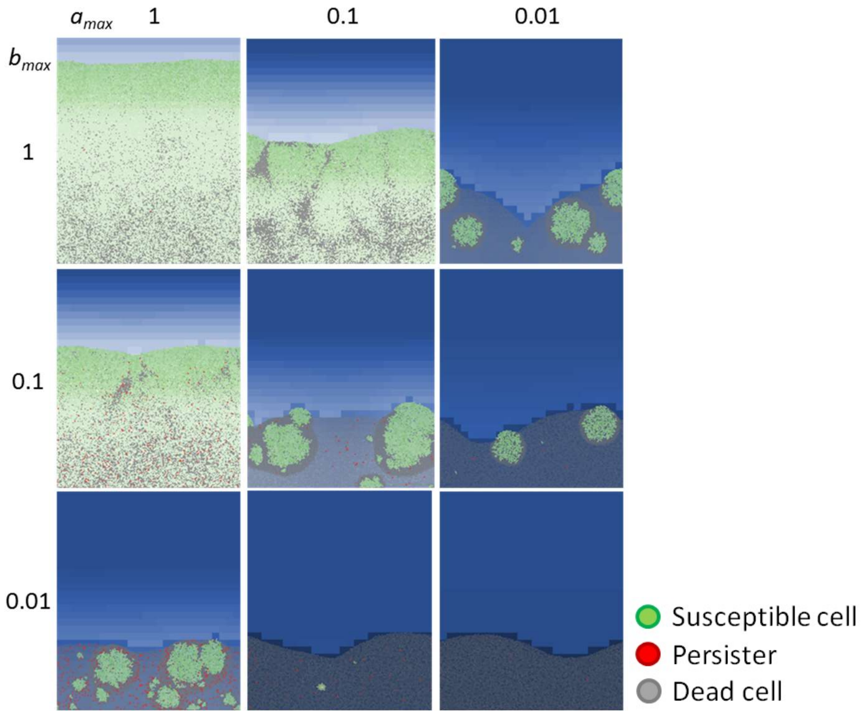

Figure S 13: Biofilms initially treated during two hours, antibiotic-dependent switches.

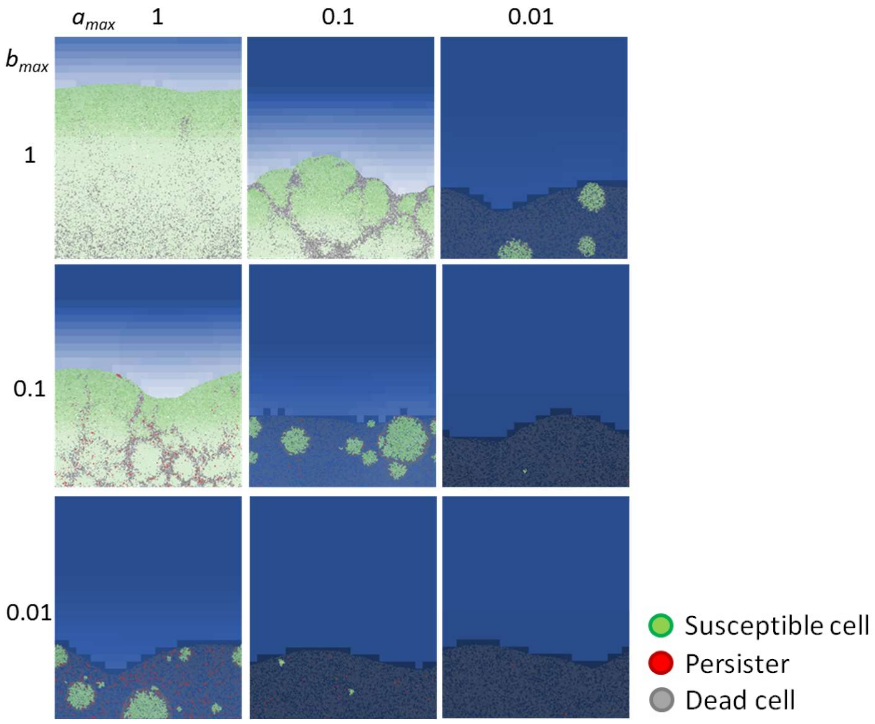

Figure S 14: Biofilms initially treated during eight hours, antibiotic-dependent switches.

7. Local sensibility analysis

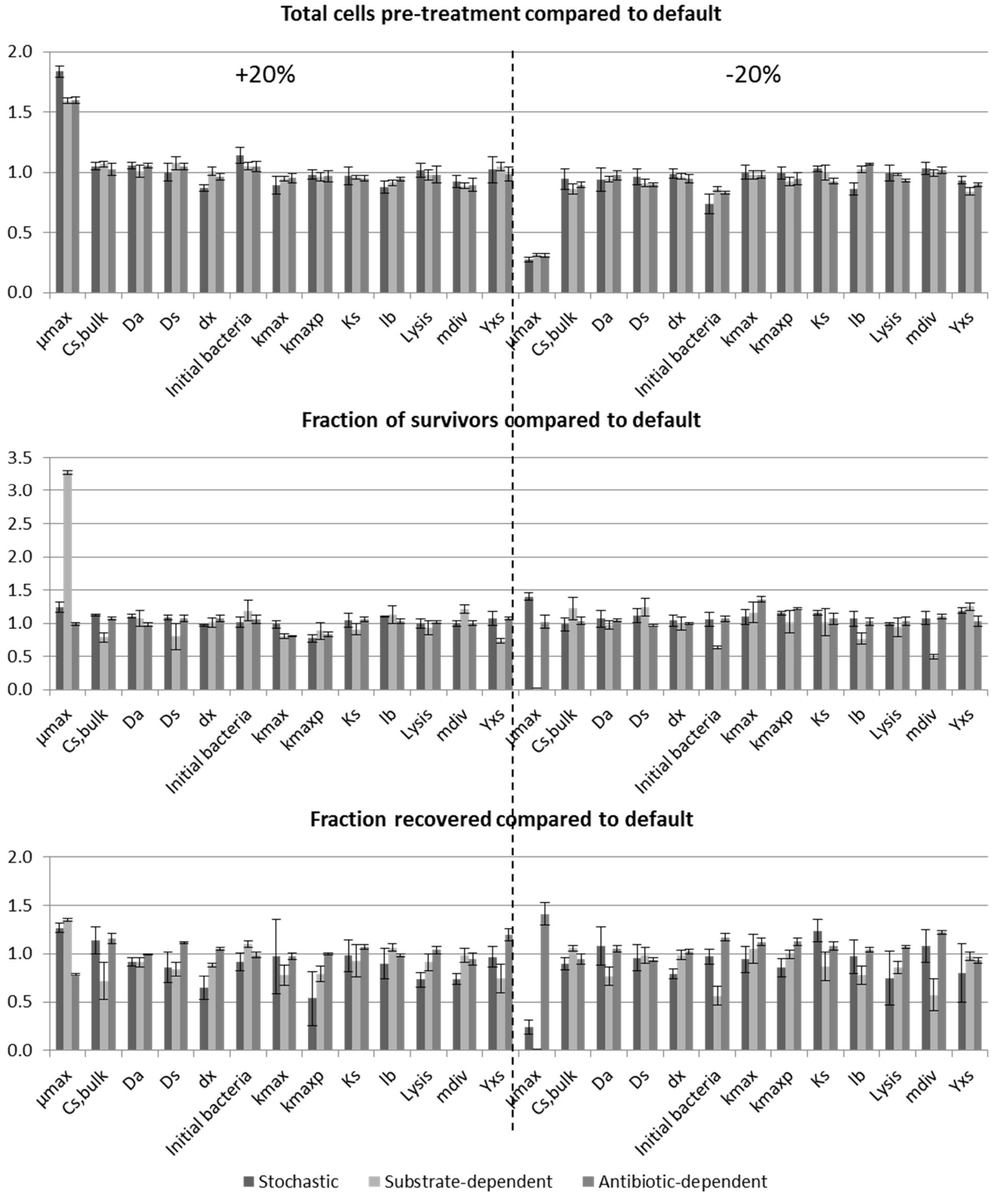

Figure S 15: Ratios of the total cells before treatment, fraction of survivors and fraction recovered compared to the default values (parameters in table S1). Each parameter has been separately increased or decreased by twenty percent compared to its default value. Results are the mean and standard deviation of three simulations.

8. Competition simulations

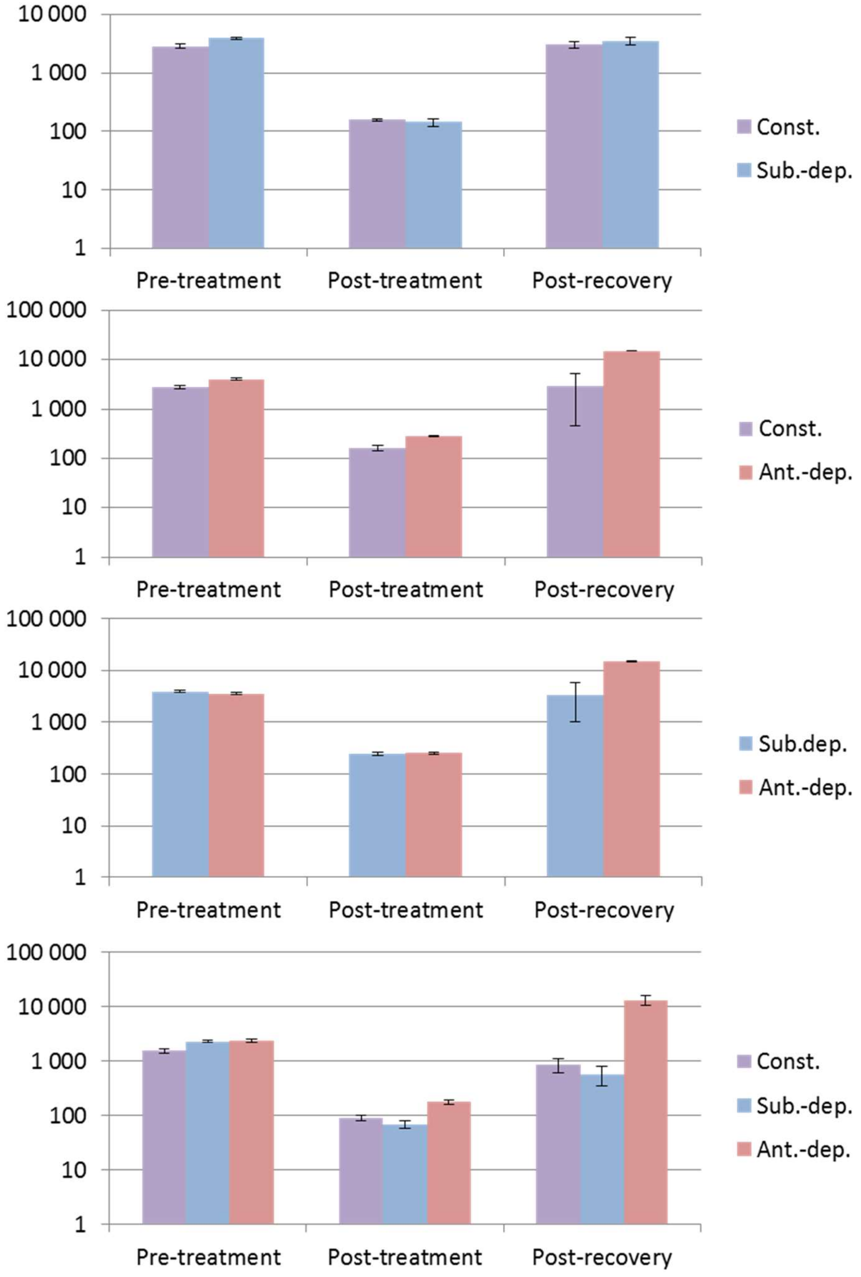

Figure S 16: Simulations of competition between strategies. Simulations with two strategies were initialized with five bacteria using each strategy. Simulations with the three strategies were initialized with three bacteria using each strategy. The parameter sets used were a_{max}=0.1 and b_{max}=0.1 for the constant strategy, a_{max}=0.1 and b_{max}=0.1 for the substrate-dependent strategy, a_{max}=0.1 and b_{max}=0.1 for the antibiotic-dependent strategy. Results are the mean and standard deviation of eight simulations with a treatment of two hours.

9. References

1. Lardon, L. a. *et al.* iDynoMiCS: Next-generation individual-based modelling of biofilms. *Environ. Microbiol.* **13,** 2416–2434 (2011).

2. Suhaimi, H., Wang, S. & Das, D. B. Glucose diffusivity in cell culture medium. *Chem. Eng. J.* **269,** 323–327 (2015).

3. Carvalho, G., Guilhen, C., Balestrino, D., Forestier, C. & Mathias, J.-D. Relating switching rates between normal and persister cells to substrate and antibiotic concentrations: a mathematical modeling approach supported by experiments. *Microb. Biotechnol.* **0,** 1–12 (2017).

4. Klima, J. & Psenner, R. Determination of Bacterial Cell Dry Mass by Transmission Electron Microscopy and Densitometric Image Analysis Determination of Bacterial Cell Dry Mass by Transmission Electron Microscopy and Densitometric Image Analysis. **64,** 688–694 (1998).
